# Sharing Cancer Survivorship Care between Oncology and Primary Care Providers: A Qualitative Study of Health Care Professionals’ Experiences

**DOI:** 10.3390/jcm9092991

**Published:** 2020-09-16

**Authors:** Karolina Lisy, Jennifer Kent, Jodi Dumbrell, Helana Kelly, Amanda Piper, Michael Jefford

**Affiliations:** 1 Department of Cancer Experiences Research, Peter MacCallum Cancer Centre, Melbourne, VIC 3000, Australia; michael.jefford@petermac.org; 2 Australian Cancer Survivorship Centre, Peter MacCallum Cancer Centre, Melbourne, VIC 3000, Australia; Jenn.Kent@nwmphn.org.au (J.K.); J.Dumbrell@alfred.org.au (J.D.); helana.kelly@petermac.org (H.K.); Amanda.Piper@cancervic.org.au (A.P.); 3 Sir Peter MacCallum Department of Oncology, The University of Melbourne, Parkville, VIC 3010, Australia

**Keywords:** cancer, survivorship, models of care, primary care, health services research

## Abstract

Survivorship care that is shared between oncology and primary care providers may be a suitable model to effectively and efficiently care for the growing survivor population, however recommendations supporting implementation are lacking. This qualitative study aimed to explore health care professionals’ (HCPs) perceived facilitators and barriers to the implementation, delivery and sustainability of shared survivorship care. Data were collected via semi-structured focus groups and analysed by inductive thematic analysis. Results identified four overarching themes: (1) considerations for HCPs; (2) considerations regarding patients; (3) considerations for planning and process; and (4) policy implications. For HCPs, subthemes included general practitioner (GP, primary care physician) knowledge and need for further training, having clear protocols for follow-up, and direct communication channels between providers. Patient considerations included identifying patients suitable for shared care, discussing shared care with patients early in their cancer journey, and patients’ relationships with their GPs. Regarding process, subthemes included rapid referral pathways back to hospital, care coordination, and ongoing data collection to inform refinement of a dynamic model. Finally, policy implications included development of policy to support a consistent shared care model, and reliable and sustainable funding mechanisms. Based on study findings, a set of recommendations for practice and policy were developed.

## 1. Introduction

In 2018, there were an estimated 18.1 million new cancer diagnoses worldwide [1]. Increased cancer incidence, together with improved cancer treatments and the ageing of the population is leading to a growing population of people living with and beyond cancer—cancer survivors. In the United States, there were an estimated 16.9 million people living with cancer on 1 January 2019; this number is estimated to rise to 22.1 million by 2030 [2]. The survivor population is similarly increasing in Australia. In 2014, 1.1 million survivors were estimated to be living in Australia, with this number projected to increase to 1.9 million by 2040 [3].

The range of issues that cancer survivors may experience has been well documented [4,5]. Research indicates that current models of oncology-led survivorship care are not meeting survivors’ needs [4,5,6,7]. Hospital-based follow-up typically focuses on surveillance and detection of recurrence or new cancers, with less attention afforded to the whole-of-person needs of patients [8,9]. Unsurprisingly, people living with and beyond cancer experience various unmet care needs across physical, psychosocial and supportive care domains [7]. An increasing number of older cancer survivors, who are more likely to have one or more comorbid conditions, adds additional complexity to the care required [10]. Furthermore, oncologist-led models are not sustainable in the context of limited health resources and increasing costs of care [6]. Development, evaluation and implementation of alternative models of care are therefore urgent priorities to effectively and efficiently care for the growing cancer survivor population. Internationally, there have been calls for an increased role for primary care in cancer follow-up [6,11].

Consensus on how primary care will fit with current models has not been reached [12]. Shared survivorship care between hospital-based and primary care providers is one model that has been investigated [13,14,15]. The first randomised controlled trial (RCT) to investigate shared care was the ProCare trial of survivors of prostate cancer [13]. Published in 2016, outcomes of the ProCare trial were in favour of shared care, demonstrating that shared care is not inferior to usual hospital-based care across a range of patient-reported outcomes, that it is preferred by patients and that it provides cost savings [13]. A further RCT currently underway is investigating the feasibility and effectiveness of shared care for survivors of colorectal cancer [14]. In this trial, two hospital appointments are replaced with GP visits in the first 12 months of follow-up, and survivors are supported through the provision of a survivorship care plan (SCP; a document summarising diagnosis, treatment, follow-up schedule and strategies to remain well [5]) and other information resources [14].

Despite increasing evidence of effectiveness and cost-effectiveness, data regarding implementation of shared cancer care is limited [16]. Opportunity exists to explore the experiences of implementing and delivering shared care from health care professionals (HCPs) involved, and to generate practical guidance that may inform further implementation of shared care models. This study aimed to explore the perceived facilitators and barriers to implementing, delivering and sustaining shared survivorship care from the perspectives of HCPs.

## 2. Methods

This study received ethical approval from the Peter MacCallum Cancer Centre Human Research Ethics Committee in September 2018 (Project number: 18/174L).

### 2.1. Participants and Recruitment

Eligible HCPs included medical oncologists, radiation oncologists, surgeons, specialist nurses, general practitioners, practice nurses, researchers and project staff who had direct experience with shared survivorship care. The research team members had existing knowledge of HCPs involved in shared care and professional networks in this area. Eligible participants were sourced from these networks, including those involved with Victorian Cancer Survivorship Program-funded projects [17], as well as ProCare [13] and SCORE [14]. Participants were purposively sampled to provide a diverse range of views based on their role, experience and knowledge of shared care, and we sought representation (at least two participants) from the following stakeholder groups: oncologists, GPs, nurses and researchers. Eligible participants were contacted directly by members of the project team via email to invite participation.

### 2.2. Data Collection

A total of five focus groups were moderated by two researchers (K.L. and J.K.). One interview was conducted with a participant who was unable to attend a focus group. Each focus group and interview were conducted over a maximum of 1.5 h in-person between November 2018 and February 2019 at the Peter MacCallum Cancer Centr in Melbourne, Australia. To begin, moderators provided a brief background and explained the purpose of the study, and answered any questions from participants. Participants were then invited to sign consent forms and complete a demographic questionnaire. Focus groups were semi-structured and guided by a question and prompt schedule developed by the investigator team. Overall, ten questions asked about participants’ experiences and views on implementation, ongoing delivery and sustainability of shared care. Following each focus group, the two moderators debriefed to discuss the focus group and any refinements to be made to the questions, prompts, question order or overall process for subsequent focus groups. Data collection continued until the research team agreed that theme saturation had been reached.

### 2.3. Data Analysis

Focus groups and the interview were recorded and transcribed verbatim for analysis. Data were analysed using inductive thematic analysis [18], and NVivo 11 software (QSR International) was used for data analysis. Two researchers (K.L. and J.D.) independently coded ~20% of total transcripts in duplicate and met to discuss similarities and differences in coding and to reach a consensus in coding rules. Following development of the initial coding framework, two researchers (K.L. and J.D.) independently coded the remaining transcripts and regularly reviewed codes as they were developed, making agreed-upon refinements. Following coding of transcripts, one researcher (K.L.) analysed all coded data to develop themes, which were presented and discussed with the entire research team to reach consensus. Final themes are presented alongside verbatim quotes from participants to illustrate each theme. Quotes are attributed to participants according to their role to transparently report on the source of each quote.

## 3. Results

Five focus groups and one interview were conducted with a total of 22 HCPs. Participants were from oncology and primary care settings, had a variety of clinical and research roles, worked with patients with different cancer types, and had substantial healthcare experience (19 participants had ≥10 years’ experience) (Table 1). Thematic analysis yielded four overarching themes: (1) considerations for HCPs; (2) considerations regarding patients; (3) considerations for planning and process; and (4) policy implications, with 17 subthemes described below (Table 2).

### 3.1. Considerations for Health Care Professionals

#### 3.1.1. Engaging HCPs in Shared Care

A barrier to implementing shared care was a lack of interest from some HCPs in both primary care and oncology settings. Strategies to mitigate this barrier and engage HCPs were suggested. For hospital staff, this included marketing shared care models through presentation at multidisciplinary meetings (MDMs, equivalent to a tumour board meeting), distributing information brochures, sharing data demonstrating satisfaction, efficiency, and demonstrating the need for shared care by highlighting busyness of clinics (“a really big facilitator was clinical need, so just the demands on our clinic meant that there had to be some sort of innovative solution to deliver care in a more integrated way and not so hospital-centric.” GP). GP engagement activities included running GP-specific information sessions or training programs, and one-to-one relationship building through visits or phone calls, however engaging GPs may be challenging. Implementation of shared care was aided by having the support of senior clinicians to champion the model, engage HCPs and lead by example. Some oncology providers have their own preferences for how to manage follow-up with their patients, which made implementing shared follow-up a challenge (“There’s always… a specialist who’s got some different ideas about how intensive follow-up should be.” Care coordinator). Navigating provider preferences may be through an approach of “respectful persistence” (Oncologist).

#### 3.1.2. Perceptions of GP Knowledge and the Need for Further Training

Participants generally felt that GPs did not need additional training to participate in shared care and reported that “we haven’t really had GPs ask us for specific training” (Care coordinator). A few participants suggested that specific training may be needed for more complex cancer cases, or conversely that complex cases may not be suitable for shared care. Rather than focus on additional training for GPs, having clear follow-up protocols and guidelines, including GPs in a shared care team from the beginning, and including GPs in communication, such as involving GPs in MDMs (where possible), were emphasised.

#### 3.1.3. Clear Roles and Responsibilities of Providers

It is important for GPs to be clear in what their role in follow-up care is; this may be achieved by including GPs in care teams (“…GP is really engaged and knows what they’re doing and we’re all really clear about what needs to be done.” GP) or may be communicated in a form or letter to the GP (“That’s what you send out isn’t it, you send out a form in the breast service where… you’re clearly identifying what the roles of the GP are and the timeframes.” Nurse). The idea of mutual respect between primary and oncology providers was also highlighted.

#### 3.1.4. Protocols and Guidance for GPs

Having access to up-to-date, evidence-based guidelines for follow-up was a facilitator for shared care. In terms of follow-up, this included having care pathways accessible online, or inserting recommended follow-up schedules into an SCP or letter. For SCPs or referral letters, having a concise summary followed by a treatment summary and detailed information made it easier for GPs to read. It is essential that GPs have guidance regarding rapid re-access pathways back into the hospital system if a serious issue arises. Reassuring patients that rapid re-access to hospital is available and that their GP has direct contact details of the oncology team and can call if an issue arises was important. Contact details and information about rapid re-access should be documented in SCPs.

#### 3.1.5. Staff Turnover and Lack of Capacity

One threat to sustainability of a shared care model was the lack of staffing, lack of capacity and staff turnover (“At the [hospital] they’ve not been able to fill that program at all and it’s been over a year so they haven’t been able to transition because there’s no staffing.” Nurse). Staff turnover impacted shared care when key clinical staff left or changed positions, or due to routine staff rotations when shared care was not firmly embedded into usual practice. Setting up ongoing, funded roles to support shared care or embedding shared follow-up care within existing job descriptions may mitigate this barrier.

### 3.2. Considerations Regarding Patients

#### 3.2.1. Stratification of Patients Suitable for Shared Care

Not all patients are suitable for shared care, and there was an acknowledgment that stratification based on individuals’ risks, symptoms, circumstances and capacity to self-manage needed to occur (“…the patients with the complex stuff, that’s what the hospital manages.” Oncologist). These factors can change over time, therefore assessment and stratification may occur at more than one time point. Patient characteristics perceived as favourable for shared care were having localised disease, absence of persistent, complex side effects, a younger age and motivation and the capacity to self-manage. Additionally, shared care may initially be most appropriate to implement in low-risk, less complex disease settings (“Pick the easy ones.” GP). There was a suggestion that for some cancers, shared care ought to be standard (“I think we have demonstrated that it can work. Each disease is different, but prostate, colorectal, it’s the diseases with high incidence and low mortality… It would seem that with some commitment and investment the entire institution could run more smoothly.” Oncologist).

#### 3.2.2. Discussing Shared Care with Patients Early and Providing Tailored Information

When patients were prepared that their follow-up would be shared with their GP and this was an expected and standard course of action, they were more reassured about not coming in to the hospital for all of their follow-up. The idea of educating patients about shared care and the purpose of follow-up was also raised, including framing cancer care as hospital-based for a defined period of time and then moving to community-based care for the longer-term. Rather than providing large volumes of information and the same information for all patients (“How much they actually read it I’m not sure.” GP), tailoring information provision based on individual patient’s needs was indicated. Different population groups may have specific needs to be considered, for example people from culturally and linguistically diverse (CALD) backgrounds may require information in other languages or translation services. Having knowledge and ready access to available resources is helpful.

#### 3.2.3. Benefits of Shared Care and Patient Acceptance

HCPs perceived that patients were generally accepting of shared care, describing patients as “happy”, “very positive” and “supporting” of shared care. Communicating the benefits of shared care to patients may increase patient acceptability and satisfaction. Benefits for patients articulated by study participants included convenience for the patient, shorter waiting and travel times with the GP, greater continuity of care, more time with their GP, and more holistic care (“There’s no doubt that a lot of the specialists deliver really great supportive care to patients, but there’s a level of referral support that GPs can give that you just don’t get when you see a hospital specialist.” Research/project staff). Patients not spending time in hospital waiting rooms with other people on cancer treatment was another perceived benefit. For CALD patients, a further advantage was having a GP who speaks their language, or patients seeing a culturally appropriate GP.

#### 3.2.4. A Patient’s Relationship with Their GP

A facilitator for shared care was patients having a regular, trusted GP. Hospital providers needed to provide a clear direction for patients around the importance of having a trusted GP and needed to ask patients who their GP is. Challenges included young people previously seeing a family GP wanting to see a different GP, people who had recently moved residence, or people who had a bad experience with their GP and wanted to change. For patients that did not have a trusted GP, shared care may act as an impetus for them to seek a regular GP. HCPs may facilitate this by maintaining a register of GPs or recommending known GPs who have an interest in cancer care.

Patient confidence in their GP was affected if a patient perceived a delay in their cancer diagnosis or if the GP did not establish the diagnosis. There may also be the perception that GPs only see patients for mild ailments and that hospitals provide more comprehensive care (“[Sarcastically] yeah, well GPs only see coughs and colds. That’s at the patient level too.” GP). Factors that helped patient confidence was knowing that their GP was included in the cancer care team and that providers communicated regarding their care (“It also I think inspires them with confidence that people are working as a team rather than two separate entities.” GP). Patients generally gained confidence in their GP over time in shared care.

### 3.3. Considerations for Planning and Process

#### 3.3.1. Designing the Shared Care Model

The design of a shared care model or intervention may incorporate knowledge gained from past experiences or projects. This included utilising resources already developed and not “reinventing the wheel”, and leveraging experiences from other contexts (for example shared antenatal care). Before deciding on specific tools, templates or processes to support shared care, it is important to pilot them. Participants also emphasised the importance of including stakeholders from the beginning of designing a shared care model. Following co-design methodology allowed the shared care model to reflect the needs of patients and HCPs, and facilitated a greater engagement and eventual acceptance of the model by consumers and HCP stakeholders. In terms of deciding where to implement shared care, a facilitator of shared care was to implement the model within the context of a well-functioning clinic to avoid existing issues or problems (“Working with the clinics that actually function really well… that don’t have a lot of issues with patients getting lost to follow up and all kinds of process problems… just so you don’t inherent that level of complexity when you’re first learning how to implement something.” Research/project staff).

#### 3.3.2. Mechanisms for Continued Stakeholder Feedback, Evaluation and Improvement

Continual cycles of evaluation, “learning from mistakes” and refinement of shared care models are needed to ensure sustainability over time. This may include formal mechanisms of stakeholder feedback, such as surveys administered to providers or patients at single time points or longitudinally (“I routinely send out patient satisfaction surveys, just a one pager with a stamped envelope and, you know, it’s basically ‘did you find it helpful, do you have a better understanding, are you happy with shared care?’” Nurse). From the earliest stages, it is important to consider data collection to support sustainability of shared care and to build a business case. Difficulties included defining which data to collect, and ensuring accuracy of data. Accurate service usage and cost data are key in engaging clinicians and may also be leveraged to secure further funding, build a business case and embed shared care as standard.

#### 3.3.3. Adequate Staffing and Care Coordination

Administrative support is required to implement and maintain shared care. Implementing shared care was considered labour-intensive, and without adequate support, HCPs found themselves performing administrative tasks such as managing correspondence, scanning and storing documents, and scheduling appointments, which was not an efficient use of their time (“You don’t want to end up paying a senior nurse money to be doing administrative tasks.” Nurse). A care coordinator role may assist in successful implementation of shared care. The role of the care coordinator included linking patients with their HCPs, linking HCPs with each other, scheduling tests and following up results, and managing appointments and reminders according to recommended treatment and follow-up schedules. Shared care may also be facilitated by an up-front approach to booking appointments with each HCP. This may mitigate patients attending multiple redundant or unnecessary appointments with different providers.

#### 3.3.4. Rapid and Accurate Communication between HCPs

There was an emphasis on communication modes that were fast, such as email where possible, or automated recall and reminder systems, fax or slower methods such as post. Electronic systems that provided a single repository of information accessible by all and SCPs were suggested as communication tools, as was including GPs in MDMs, and for remote GPs, utilising telehealth. An important aspect of this theme was to make communication and information sharing as effortless and efficient as possible. Communication errors hindered shared care, including missing paperwork, faxes or test results. It was widely regarded as essential for GPs to have a direct phone access to the hospital, either to a specialist nurse, liaison officer or coordinator, or direct to the oncologist(s). GPs valued having a direct contact phone number and “having a person you know who you can go to” (GP).

#### 3.3.5. Electronic Medical Records and IT Systems

Electronic medical records (EMRs) that can be accessed at different sites by multiple providers may facilitate shared care by allowing input and access of medical data in real-time by all members of a patient’s care team. Given the importance of SCPs and the need for rapid information sharing, having information technology (IT) solutions that would enable automatic generation of SCPs and accessing information in real time would ease this problem. The time taken to manually develop SCPs was frequently cited as a barrier to shared care. The EMR was considered to be “a key part of the longer-term sustainability, so that you can actually automate a lot of the processes of creating a care plan, that it doesn’t take an hour to two hours to produce” (GP). Current systems were considered fragmented and inadequate.

#### 3.3.6. Allowing Time for Cultural Change

Adoption of shared care and change in process may not happen immediately; time may be needed for people to get used to a shared care model. Participants typically reported that once HCPs and patients experienced a shared care model, even if they were initially resistant, they accepted it and were supporting of its continuation. It took time for shared care to become part of their routine. Cultural change also included language used by clinicians when speaking with patients and with each other and using/normalising the terms survivorship and shared care.

### 3.4. Policy Implications

#### 3.4.1. Having Executive Support and Consistent Policy

Support for shared care at both hospital executive and government levels is an important facilitator (“You do probably need a bit of that top down stuff to say look this is now the standard of care.” GP). Lack of executive support was seen as a reason for shared care models not succeeding at single sites, and lack of government policy was thought to contribute to a lack of consistency across multiple sites. Having a consistent model of care across different hospitals would streamline the process for GPs, so GPs would receive the same information and have the same requirements regardless of where their patient was treated (“Your best model needs to be applied to all hospitals.” GP).

#### 3.4.2. Reliable and Sustainable Funding

In the context of shared care projects, funding via a grant was a clear enabler of implementing shared care. Funding was useful in developing the resources needed to support shared care and also to fund the positions required to perform the necessary tasks. However, issues with sustainability occur through this funding model. Survivorship care within primary care needs to be appropriately funded. Outside of grant funding, having aspects of survivorship care funded by government was also helpful in supporting primary care involvement.

## 4. Discussion

A shared model of survivorship care may confer benefits to patients and also health systems, particularly in light of a growing survivorship population and shortage of oncology providers. Indeed, there has been increasing attention internationally on the need to adopt alternative models of survivorship care, beginning with the seminal 2006 report “From Cancer Patient to Cancer Survivor: Lost in Transition” [5]. This report (amongst other recommendations) advocated for the exploration of new models of care and highlighted the importance of coordination between specialist and primary care providers in order to meet the holistic needs of both patients and caregivers living with and after cancer. More recent publications have built upon these early recommendations, arguing for the need to develop “personalised” care pathways, where patients may be triaged to one of several different pathways (which may include shared care) based on their care needs, risk profiles and personal circumstances [6,19,20]. In Australia, shared care has been recommended [11,20], investigated in completed and ongoing RCTs [13,14], and implemented and evaluated via funded projects in specific settings [17], however has not been widely adopted. By drawing on the expertise of HCPs who have been involved in these initiatives, the present study aimed to develop a suite of recommendations that may support broader implementation of shared care.

Qualitative enquiry of HCP experiences of shared care has yielded a rich description of both the enablers and challenges to implementing and sustaining this model within the context of cancer survivorship and led to development of recommendations to support implementation (Table 3). Themes included co-designing shared care interventions and models, establishing processes for outcome data collection and regular stakeholder feedback, implementing shared care in low-risk settings, and employing stratified patient pathways within a dynamic shared care model. Recommendations speak to these themes, and consider day-to-day processes in delivering shared care and implications for HCPs and patients. Importantly, recommendations also consider longer-term success and the creation of an environment that will support shared care through the development of policy and sustainable funding mechanisms.

There are several important contrasts between the present study and the existing literature. The authors recently conducted a systematic review of qualitative and quantitative evidence addressing facilitators and barriers to shared care for people with cancer [21]. The present study confirms many of the themes synthesized in the review and importantly adds a number of novel themes not present in the review, and to our knowledge, in the available literature. These include themes concerning the design, implementation and evaluation of the shared care model, challenges and strategies regarding HCP engagement in shared care, patients’ relationships with their GPs, and assessment and stratification of patients suitable for a shared care pathway. Furthermore, there are important differences in the perceptions of further training for GPs and the need to define the roles of each provider.

Most notably, participants in our study generally thought GPs had sufficient knowledge to care for cancer survivors and did not need additional training in order to participate in shared care. In contrast, earlier studies (as well as the systematic review) have consistently reported that GPs require additional training to provide follow-up care, and this has been reported from the perspectives of GPs [22,23], specialists [24] and patients [25,26,27]. One recent Australian study however has revealed mixed results regarding GPs’ attitudes on the need to undergo further training [28]. Our results suggest that requiring training to enable GPs to participate in shared care may act as a barrier to their involvement. Emphasis may be better placed on supporting GPs through provision of clear follow-up schedules and guidelines, which has an integral component of shared care interventions investigated to-date [13,14], including GPs as part of a shared care team, and providing GPs with direct avenues of contact with specialists when needed.

Another point of difference between the present study and other literature regards perceptions on the need for role clarity, which has been reported in other studies of shared cancer care [21,24,29] and in shared care in other contexts [30,31]. While clarifying and documenting the respective roles and responsibilities of oncology and primary care providers did arise in the current study, the emphasis was on mutual respect between providers and ensuring patients understood which HCP to seek help from for issues that might arise, rather than delineating boundaries of GP responsibilities. There was also greater emphasis on GPs having access to the necessary information to allow them to care for their patients, including diagnostic and treatment summaries and a survivorship care plan detailing follow-up care. Due to the differences in perceptions of role clarity and GP training between ours and other studies, these may represent an avenue for further research and recommendations.

### Strengths and Limitations

Although this study includes a range of perspectives, including those of GPs, oncologists, specialist and practice nurses, and researchers, it did not include the patient perspective. Future research may ascertain factors affecting implementation, delivery and sustainability of shared care from the perspective of the survivors who experience it. A number of study participants were identified based on their involvement in known RCTs investigating shared care. It is possible that their experiences of shared care may be more favourable due to their interest in these research studies. Given the current international interest in redesigning models of survivorship care [6,11], study results are likely to be of interest to clinicians and policymakers internationally. However, as we investigated the experiences of HCPs located in Victoria, Australia, some findings and implications for practice may not be applicable or relevant to international settings, particularly where health systems and funding mechanisms vary substantially from Australia.

## Figures and Tables

**Table 1:** Characteristics of study participants.

Characteristic	*n*
**Role**	
Nurse	8
GP	4
Care coordinator	2
Research/project staff	6
Oncologist	2
**Clinical setting**	
Breast	6
Colorectal	3
Gynaecological	2
Prostate	1
Haematology	1
Combination/any	9
**Length of experience**	
<10 years	3
10–20 years	5
21–30 years	10
>31 years	4
**TOTAL**	22

GP: general practitioner.

**Table 2:** Summary of themes and subthemes.

Theme/Subtheme	Example Participant Quotes
Considerations for Health Care Professionals	
Engaging HCPs in shared care	“…the surgeons and the oncologists have been motivated by… well actually both have been motivated by horrendously busy clinics so they’ve got an inherent sort of time stress motivation.” (Care coordinator) “Getting GP engagement is the biggest issue that we had. Like we held multiple forums and hardly anyone would attend. So I kind of walked into this very apprehensive, this GP shared care.” (Nurse) “We’ve really engaged some champion doctors that’s been wonderful, so we have some oncologists that are brilliant at referring to us, and also increasingly surgeons that are becoming far more initiators of referrals. And there is a new young surgeon at the MDM * who goes ‘would that patient be appropriate for shared care?’ which is great, it’s no longer us doing it and that was like a yay moment, that was really good.” (Research/project staff) “I think some of the barriers can sometimes be, you know, clinicians not really comfortable with changing the way things are done. And I found in… probably in particular medical oncologists were the ones that wanted to keep holding onto the patients.” (Nurse)
Perceptions of GP knowledge and the need for further training	“The assumption that GPs need to go and have all this training, I don’t really support that model. I think a lot is about tapping into a lot of their existing skills already and just providing very clear guidance about that individual patient that is there when they need it. And then GPs make use of that very specific information very well, they don’t need necessarily to go to lots of workshops… People can choose to upskill but I don’t think it’s a prerequisite.” (GP) “…we’ve had a really strong message that GPs actually already know a lot about surveillance in the bowel cancer space and don’t really need to be told sort of some of the 101s of how to examine a patient.” (Research/project staff) “…because what you should ask them to do shouldn’t be so complicated that they have to go onto a training course, ‘cause if it is that complicated they possibly shouldn’t be seeing the GP for shared care.” (GP) “If the GP could be involved at the very beginning of the whole process where the diagnosis has been made and plans, somehow for the MDT *… the GP will feel more empowered and know what’s going on and who the clinicians are.” (GP)
Clarity of roles and responsibilities of providers	“I think the philosophy is mutual respect, because I think there is nothing that will disengage GPs more than being lectured to by the folk in the ivory tower who don’t actually know how hard it is to do general practice and therefore I think we seriously need to respect... And, you know, the idea of role clarity can sort of sound good but it can get… it can very quickly morph into, you know, stick to your knitting, anything that’s hard, you know, you’re not good enough to make these hard decisions, and we stay away from that.” (Oncologist) “And the shared care needs to be… we need to know what’s been… what’s the treatment, what are the side effects and what’s being monitored by them and what are we doing, what’s our role.” (GP)
Protocols and guidance for GPs	“I think the guidelines have actually been quite clear, and that was one of the things that we received feedback from from one of our GPs who said it’s actually quite clear what you’re requiring of us.” (Nurse) “And for the GP it has to be easy, I mean we’re seeing so much coming through so it has to be sort of OK, come in and we’re doing this, this, this, fine, yeah, check, bang, bang.” (GP) “They’re reworking their shared care plan and the fact that at the top it’s the action points, the summary first and then the detail… So maybe looking at a thing that could make shared care work is concise information that is easy for the GP to read and action” (GP) “I think that’s the most important thing to get buy in from the patients is that they need to know if they get a problem or a new situation, a new breast lump or a symptom, they can get rapid access back…” (Nurse) “It should be though documented in their care plan shouldn’t it the rapid access” (Research/project staff)
Staff turnover and lack of capacity	“Something very basic like the rotating doctors, again a new Fellow and a new Registrar every year and we’ve got to teach them all over again… ‘cause it’s not built into the systems to handover to the new doctors that that’s what’s happening now.” (Care coordinator) “Well unfortunately the model didn’t continue at [hospital]. So it was dependent on the nurse consultant to do it. When I left [hospital] they decided not to replace the nurse consultant role. So the clinic closed so people are back in five year follow-up. So unfortunately, you know, it became very dependent on one clinician doing it… [there] needed to be multiple people being taught how to do the clinic and it didn’t happen that way.” (Nurse)
**Considerations Regarding Patients**	
Stratification of patients suitable for shared care	“With your identification of your women you’d be doing some type of stratification to identify who’s suitable with regard to capacity to do… to be empowered and to self-manage in that respect, I imagine.” (Nurse) “Risk stratification applies to all survivorship doesn’t it? I mean when you’ve got someone who’s, um, you know, high risk of recurrence or developing secondaries and needs that sort of perhaps specialist monitoring then, you know, you think twice about shared care, are they really appropriate for it.” (Research/project staff) “Some patients who are very symptomatic at end of adjuvant treatment and having a really rough time, where the oncologists are like nah we can’t discharge them to shared care model just yet, or well it’s not discharged but, you know. But three months or six months later they’re saying can you put this person on shared care. So we’ve also had some patients who said I don’t want shared care and then they have a taste of regular follow up and they go actually I’m ready for shared care. Yeah so I think it’s a bit of a movable feast.” (Research/project staff)
Discussing shared care with patients early and providing tailored information	“That’s really important, yeah, because it can be quite a… it’s like a separation for the patient… and they need to be prepared that, you know, at what point their care will shift into either a shared care model or an entirely primary care level.” (Care coordinator) “So we’re doing that but I think we over-provide… a lot of people if they get a box and they probably say ‘oh yeah I do have a little bit of problem with feeling tired sometimes’, but it doesn’t necessarily mean that they all need the Cancer Council fatigue booklet.” (Nurse) “I think for us we would flip it around a bit so that showbag of information or folder of information, I think we would invest some time in peeling that back a bit, and so instead of taking that kind of here take everything…here’s what we think you need.” (Nurse) “Having those resources available for our use, so evidence-based resources is really helpful… if they don’t want a care plan that we can give them something else.” (Nurse)
Benefits of shared care and patient acceptance	“You’ve got the patient feedback, so we were constantly reviewing patients’ experiences which were overwhelmingly positive, you know, they liked the fact that they, you know, didn’t have to pay for parking, they could just go to their local GP, they didn’t have to take a day off work, so all those factors. So there were far more benefits to it than negatives.” (Nurse) “There’s no doubt that a lot of the specialists deliver really great supportive care to patients, but there’s a level of referral support that GPs can give that you just don’t get when you see a hospital specialist and a very holistic approach.” (Care coordinator) “We’ve had a lot of patients of non-English speaking background, and often it was better ‘cause often their GP speaks their language and already has a good connection. So I think if anything shared care often works better rather than coming to the hospital with an interpreter.” (Nurse)
A patient’s relationship with their GP	“Even patients who, you know, it was their GP who first noticed something was wrong, their confidence level in that particular GP is a lot higher than those who, you know, are concerned why their GP didn’t pick it up in the first place.” (Research/project staff) “If you’ve got a long-term relationship with your GP your ability to talk really openly with the GP about really personal things, and actually just the quality of that relationship and the level of trust. And also for people who have one GP, I think people can find that really powerful having one person, whereas at the hospital they might like all their clinicians but they get someone different, you know, every few weeks or few months or whatever.” (Care coordinator) “We’ve actually got on our database, and it was from the GPs that attended our information seminars, you know, those that we’ve… champions in the community for shared care as well. So if you’ve got a patient who says do you have anyone in mind…” (Nurse)
**Considerations for Planning and Process**	
Designing the shared care model	“what we need to do though is break it down in terms of what can we do but more importantly what can we scale so that we’re not reinventing processes and finding things that work in one space and scaling them to other spaces.” (Oncologist) “We had consumers and specialists and GP reps meet, and some GPs, to really design those things. And I think that was helpful.” (Research/project staff) “So certainly having stakeholders who really had an invested interest in wanting this to work. And it had to be multidisciplinary in that perspective, it certainly had to include consumer reps as well. We had very regular meetings over quite a number of years actually, probably three, four years at least, and that included management as well as medical, nursing, consumer, GP liaison reps.” (Nurse)
Mechanisms for continued stakeholder feedback, evaluation and improvement	“We were really eager to I guess get some feedback about what our consumers felt, was this a valuable interaction and what they thought… So we’ve set in place some regular feedback marks where we get that consumer feedback and engagement. And with the GPs as well.” (Nurse) “I think having access to good data is important and the quality of your outpatient utilisation data’s really important. And I must say at both the sites I work at there’s some real problems with the accuracy of that data, and there are clinics where there’s template issues, so the level of demand is not… is really understated by that data.” (Care coordinator) “Right from the start when you’re implementing these kind of things you need to be thinking about that long-term sustainability and gathering that data and building your case and keeping those updates at the MDM * and utilising your champions and spreading the word to, yeah, to help drive that and help keep that momentum up and build that business case around it.” (Nurse)
Adequate staffing and care coordination	“You need a fairly high functioning admin person to be able to put those [survivorship care plans] together, so that is one of the challenges, yeah.” (Nurse) “The admin is… it’s crucial. I’m really lucky that I have sort of an admin assistant who does a lot of the mail outs and then, you know, we can get really good, um, strike rate with sending out our questionnaires and getting our screening tools back and she organises all of that, so it really is like a well-oiled machine and we’re able to allocate appointments.” (Nurse) “There’s no way we could’ve established the model without a care coordinator person, there’s absolutely no way we could’ve done it, so that was, you know, it didn’t just assist, it was absolutely critical.” (Research/project staff)
Rapid and accurate communication between HCPs	“We do snail mail unfortunately” (Nurse) “I think some of the feedback from our GP sessions was, um, they don’t want so much paperwork. You know, if we could just encrypt it and send it electronically, in an ideal world I think that would be fantastic.” (Nurse) “We’re trying to help the GPs, there is an awful lot on ‘here are ten ways to get in touch with us and feel free to do so’. You know, there’s an email that comes to me and [name] sees it… there are fax numbers, here’s a phone number, call the registrar, and in the end my name’s signed there and people can phone me.” (Oncologist) “Very importantly they also have to feel that they can quickly get advice if needed.” (GP)
Electronic medical records and IT systems	“And again IT, easy access, ready access for clinicians when they’re seeing patients, or perhaps GPs as well ‘cause I don’t know how ready access their viewing of the care plans are either.” (Nurse) “What they haven’t done though is worked out those fundamental interfaces in data sharing and governance and ethics, you know. So I’m a very positive and forward focused looking person but I think there are gonna be challenges with the EMR * that won’t overcome it. At the moment the EMRs don’t interface properly with iPMs [patient information management systems] and radiology and pathology let alone getting them to interface with GPs and other service providers. But if they can overcome that I’m hopeful that it could but there’s going to be challenges at a fundamental level.” (Oncologist)
Allowing time for cultural change	“It’s probably just taken time and quite a few knockbacks with some people and then they’ve just come on board over time, and I think some clinicians just need to see it working with other clinicians first and then they’ll give it a go.” (Nurse)
**Policy Implications**	
Having executive support and consistent policy	“…we really need more than dollars don’t we… we need the engagement from those key stakeholders to allow us to pursue those strategies that will enable it, and they’re huge constraints. We need government to get on board, we need policymakers to get on board.” (Oncologist) “I think the model that we have put together is readily applicable to a number of tumour streams… but I’ve been disappointed that others haven’t sort of come along and taken a look… I think that’s fallen over because of lack of ongoing hospital support.” (Oncologist) “Another thing that worked well on the [name] shared care project is the executive support. So there’s support from the hospital saying yeah we want to do this.” (Research/project staff) “I think perhaps part of the problem is that we’re all at different stages and, you know, if the Federal government said right, now every service has to do this it would’ve been universal and everyone would’ve understood. Whereas I think it’s come in in dribs and drabs, different approaches and different models of care plans and everyone’s reinventing the wheel. And not necessarily reinventing but doing their own version of the wheel.” (Nurse) “We need a cultural approach to this, everyone doing the same thing, working at the same cadence, taking processes that work and adopting them across the board.” (Oncologist) “And as you say different hospitals have sort of different care plans, so the same thing, so to keep the hospitals consistent too so that someone from [hospital A] is not doing something different to [hospital B] or… as the GP it would be helpful not getting different messages.” (GP)
Reliable and sustainable funding	“So if you’re working in a tumour stream where there’s traditionally not a care coordinator, um, I think you’ve got a massive challenge at actually how you’re going to fund a new position, and also how you’re going to fund some administrative support for that position.” (Research/project staff) “But I think in general practice there are enough item numbers to, um, just change it a little bit to make it actually worthwhile for the GPs, which I think at the moment we’re actually halfway there, a lot, you know, I think personally a lot ahead than the hospitals in terms of funding for shared care.” (GP) “…so what you’re hearing is clinical champions that lose their source of income and the person leaves. So if you can find a way to create sustainable job descriptions that fit those needs for the government that would be huge I reckon.” (Oncologist)

* MDT: multidisciplinary team; MDM: multidisciplinary meeting (equivalent to a tumor board meeting); EMR: electronic medical record. Themes are indicated in bold text.

**Table 3:** Implications for practice and policy.

**Implications for health care professionals**	Facilitate engagement of both specialists and GPs with shared care by: promoting the benefits of shared care, such as more comprehensive, holistic care, easing the pressure on busy clinics, sharing data regarding improved efficiency, presentation at MDMs for specialists; running information sessions, engaging in GP placements, one-to-one relationship building for GPs. Identify a senior clinical lead to act as a shared care champion, provide leadership and set an example for other clinicians. Involve GPs as part of the shared care team from the point of diagnosis onwards, and where feasible, including the GP in MDMs. Have additional training for GPs available but not required; requiring GPs to undergo training may be a barrier to participation in shared care. Ensure that GPs have clear information about patients’ diagnoses, treatment history, expected side effects, follow-up plan and need for urgent review—this may be in a letter or SCP.Provide clear and concise guidance to GPs regarding their role in follow-up, with a summary of key information including timelines and actions required. These must include re-entry procedures if recurrence is suspected. Provide a direct line of communication (direct phone number or email address) between primary care and hospital-based providers. Consider a dedicated care coordinator role to enable shared care, including scheduling appointments, generating SCPs, advising patients, facilitating communication between different providers and between providers and patients etc.
**Implications for patients**	Stratify patients based on risk of recurrence or new cancers, persistent, complex side effects, personal circumstances and capacity for self-management. This may involve re-assessment of patients over time and re-stratification to a model of care. Discuss shared care with patients early so they know to expect shared care and consider this standard. Enquire about a patient’s GP early. If a patient does not have a named, trusted GP, it may be useful to suggest a GP known to have an interest in cancer care. Engage patients in shared care by promoting and communicating the benefits of shared follow-up, such as more comprehensive, holistic care, reduced travel and waiting times and greater continuity of care with their GP. Educate patients around the role of the GP in their follow-up care, and on which HCP to see the different issues they may experience. Provide information resources to patients but avoid overwhelming patients with too much information. Critical information to provide to patients includes contact details for their providers. Consider the needs of different patient groups (for example based on language spoken or cultural background) and availability of suitable resources when planning shared care.
**Implications for planning and process**	Initially, implement shared care in clinics that function well and in less complex, low risk cancer settings. Adopt a co-design approach (with both patients and HCPs) to facilitate greater engagement with shared care and ensure stakeholder needs are being met. Pilot all tools and processes before committing to a shared care model. Determine appropriate health service and patient outcome measures and processes for accurate data collection for longitudinal evaluation of shared care. Where possible, establish well-functioning cross-sector IT systems. IT solutions may facilitate communication between providers in real-time (for example through an EMR) and generation of SCPs. Establish rapid referral pathways to specialist providers if recurrence or other serious events are suspected. Rapid re-access procedures should be clearly documented in an SCP and communicated with patients. Consider ongoing mechanisms of stakeholder feedback or evaluation measures to facilitate continual improvement and refinement of the shared care model. Consider establishing and maintaining a register of GPs interested in cancer care; this may be helpful to refer patients without a known, trusted GP.
**Implications for policy**	Advocate for a policy environment that supports shared care as standard care. Establish a consistent policy regarding shared care and SCPs to create standardised care and reduce variation across settings. Advocate for sustainable funding mechanisms to support shared care.

EMR: electronic medical record; GP: general practitioner; IT: information technology: MDM: multidisciplinary meeting (equivalent to a tumor board meeting); SCP: survivorship care plan.
